# Reversing Gray Hair: Inspiring the Development of New Therapies Through Research on Hair Pigmentation and Repigmentation Progress

**DOI:** 10.7150/ijbs.86911

**Published:** 2023-08-28

**Authors:** Zhaorui Feng, Yi Qin, Guan Jiang

**Affiliations:** 1 Department of Dermatology, Affiliated Hospital of Xuzhou Medical University, Xuzhou, China.; 2 Department of Dermatology, Xuzhou Medical University, Xuzhou, China.

**Keywords:** hair repigmentation, hair pigmentation, melanogenesis, targeting drugs, topical treatments

## Abstract

Hair graying is a common and visible sign of aging resulting from decreased or absence of melanogenesis. Although it has been established that gray hair greatly impacts people's mental health and social life, there is no effective countermeasure other than hair dyes. It has long been thought that reversal of gray hair on a large scale is rare. However, a recent study reported that individual gray hair darkening is a common phenomenon, suggesting the possibility of large-scale reversal of gray hair. In this article, we summarize the regulation mechanism of melanogenesis and review existing cases of hair repigmentation caused by several factors, including monoclonal antibodies drugs, tyrosine kinase inhibitors (TKIs), immunomodulators, other drugs, micro-injury, and tumors, and speculate on the mechanisms behind them. This review offers some insights for further research into the modulation of melanogenesis and presents a novel perspective on the development of clinical therapies, with emphasis on topical treatments.

## Introduction

Hair graying is a common, visible, and early marker of human aging 1, 2. It is now understood that human hair and its pigmentation can greatly affect societal perception, emotional well-being, and psychological state 3. Considering the potential health risks posed by hair dyes 4, new strategies for hair color change are warranted.

Hair graying has long been thought of as an irreversible age-related process. Nonetheless, recent research has revealed that restoring the color of a single gray hair to its original pigmentation is a general phenomenon regardless of age, gender, ethnicity, and corporeal regions but only appears in a single anagen of rare HFs 5. Although there is great heterogeneity between hair follicles (HF), the similarities between the processes of graying and repigmentation imply the potential for systemic behavioral factors (such as life stress) to simultaneously regulate the pigmentation of multiple HFs. Meanwhile, proteomics and computational simulation have proven the theoretical possibility of reversing gray hair temporarily 5. Based on the evidence presented, it becomes evident that the prevention or reversal of hair graying holds significant promise for the future. Therefore, in this review, we summarize the regulation of melanogenesis and focus on cases of hair repigmentation and the mechanisms behind them, trying to inspire future research on the regulation of melanogenesis and therapy development.

## Overview of hair pigmentation and hair graying

Melanocytes in human HF are classified into several sub-populations according to function, differentiation status, and location. Within anagen hair follicles, melanocytes responsible for hair pigmentation primarily reside in the hair matrix surrounding the mid to upper dermal papilla. These bulbar melanocytes express active tyrosinase and the melanogenic intermediate dihydroxyphenylalanine (DOPA) and are considered a component of hair follicle pigment unit (HFPU) 1, 6. Melanogenesis occurs in specialized lysosomal-related organelles termed melanosomes. The melanin-containing melanosomes are then transferred to the keratinocytes of hair shaft through dendritic and filopodial processes 7. Melanocyte stem cells (McSCs) are located in the bulge and the sub-bulge area of the outer root sheath (ORS). These cells are immature and poorly or un-pigmented 6. Recent studies have indicated that the majority of melanocyte stem cells possess a unique and unexpected mechanism for self-renewal and melanogenic melanocyte production. These McSCs exhibit a distinctive ability to switch between transit-amplifying and stem cell stages, which fundamentally distinguishes them from other self-renewing systems 8. According to live imaging and single-cell RNA sequencing, McSCs move between the transit-amplifying and hair follicle stem cell compartments through dedifferentiation, reversibly entering multiple differentiation stages controlled by the local microenvironment. Long-term lineage tracing studies have provided compelling evidence that the sustained melanocyte stem cell system is supported by reverted McSCs that dedifferentiate from transit-amplifying stage rather than reserved population of stem cells that inherently maintained in an undifferentiated state.

Indeed, hair pigmentation and the hair cycle are inextricably linked. The hair cycle consists of three distinct stages: anagen, catagen, and telogen. Hair pigmentation only happens during anagen because the melanogenic HFPU exists in this period 9-11. Most differentiated melanocytes experience apoptosis in the catagen phase, while bulge McSCs survive in the secondary hair germ 12-14. As a new anagen phase is initiated, the surviving McSCs differentiate into melanogenic melanocytes to rebuild the HFPU 13, 15.

Current evidence suggests that multiple factors can influence the process of hair graying 6. However, the root cause of hair graying is the dysfunction and cell death of melanogenic melanocytes in the HFPU. It is widely thought that during a single anagen phase, the HFPU is self-maintained and does not require replenishment from McSCs 1. Therefore, the initial onset of hair graying is not necessarily related to the depletion of McSCs. However, as individuals age, stranded McSCs accumulate over time and do not contribute to the production of mature melanocytes 8, 16, 17. The preservation of McSCs is crucial for the reconstruction of the HFPU and provides the possibility for the reversal of grey hair. Once McSCs are exhausted, hair graying becomes irreversible 1.

Epithelial stem cells (EpSCs) in the hair follicle are crucial in providing a functional niche for melanocyte stem cells 18, 19. The offspring of EpSCs in the HF bulge and hair germ develop into outer root sheath (ORS) and transit-amplifying cells (TACs) in the HF matrix, which support HF regeneration. The TACs differentiate into several lineages that eventually give rise to the hair shaft and its supporting components 20. Recently, single-cell transcriptomics revealed that P53 pathway activation-induced specific depletion of matrix TAC, but not HFSCs, is associated with early-stage human hair graying 20. Therefore, the effects of regulatory factors on cells within the HF, other than melanocytes, are also considered in this context.

It is crucial to note that even visually colorless scalp HFs may still have a few hair bulb melanocytes. Some may even continue to produce melanin, although they lack dendritic morphology and melanin transmission to the hair shaft 21. Thus, it is theoretically possible that a special therapy that reverses hair graying before all the hair bulb melanocytes and McSCs disappear can be developed in the future.

## Signaling pathways in the regulation of melanogenesis

### Wnt/β-catenin signaling pathway

When Wnt molecules bind to their receptors, β-catenin is activated, increasing Melanocyte Inducing Transcription Factor (MITF) transcription in McSCs. Glycogen synthase kinase 3β (GSK3β) phosphorylates β-catenin without Wnt signaling, which causes it to break down through a proteasome-dependent mechanism. The activation of Wnt signaling also increases Endothelin Receptor Type B (EDNRB) signaling 22, 23. These effects of Wnt signaling synergistically promote MsSCs' migration, proliferation, differentiation, and melanogenesis. Moreover, simultaneous activation of Wnt/β-catenin signaling in EpSCs and McSCs initiates pigmented hair regeneration 24.

### MC1R

As one of the central regulators of pigmentation, Melanocortin 1 Receptor (MC1R) signaling promotes melanogenesis and melanosome transfer in melanocytes. Interestingly, it has been shown that ultraviolet B (UVB) induces keratinocytes to express pro-opiomelanocortin (POMC), which is cleaved to release α-melanocyte-stimulating hormone (α-MSH) and adrenocorticotropic hormone (ACTH), both ligands of MC1R 25. MC1R activates adenylyl cyclase and then increases cyclic adenosine monophosphate(cAMP). One of the numerous effects of cAMP, mediated by cAMP-dependent protein kinase A (PKA), is the phosphorylation of cAMP-responsive element-binding protein (CREB), which stimulates MITF transcription 26.

Store‐operated Ca2+ entry (SOCE) is initiated upon endoplasmic reticulum (ER) Ca2+ release. The Ca2+ binding ER membrane protein stromal interaction molecule 1 (STIM1) senses the Ca2+ store depletion and then oligomerizes and interacts with plasma membrane store-operated Ca2+ (SOC) channel Orai1, leading to Ca2+ influx 27. α-MSH-induced cAMP stimulates ER Ca2+ release through phospholipase C (PLC)/ inositol triphosphate (IP3) signaling. Subsequently, STIM1 activates the plasma membrane-localized adenylyl cyclase 6 (ADCY6), which acts independently of Orai1, to increase cAMP. This positive feedback loop controlled by cAMP‐Ca2+ crosstalk further promotes the effects of α-MSH 28.

In addition to promoting melanogenesis in melanocytes, the activation of MC1R also enhances the repair of Ultraviolet radiation (UVR)-induced DNA damage 29. Besides, it has been reported that injury or UVB-induced epidermal migration of McSCs also relies on MC1R signaling 30.

### SCF/C-KIT

The stem cell factor (SCF)/tyrosine kinase receptor (KIT) signaling system plays a significant role in melanogenesis. When SCF binds to its receptor c-KIT, it triggers the receptor's tyrosine kinase activity, which results in receptor phosphorylation. As a result of the phosphorylation of c-KIT, mitogen-activated protein kinase (MAPK) is stimulated and then triggers the phosphorylation of CREB, activating MITF 31. An extracellular signal-regulated kinase (ERK) can be activated by c-KIT signaling. While ERK signaling activates CREB to promote melanogenesis, it has also been shown to phosphorylate MITF, leading to its ubiquitination and subsequent degradation, thus forming a feedback loop in melanin regulation 32. However, it cannot be ignored that the presence of MITF alone is not sufficient for Tyr expression and that KIT signaling is needed not only for the proliferation and survival of melanoblasts but also for Tyr induction and the transition of melanoblasts to mature melanocytes 33.

### EDN/EDNRB

Endothelin receptor B (EDNRB) has been identified as playing indispensable roles in the maintenance, proliferation, differentiation, and migration of McSCs 22. Besides, EDNRB signaling activates PLC/IP3/ signaling and ER Ca2+ release 27. Following SOCE, MITF expression significantly increases via the Ca2+/PKC/MAPK/p90 ribosomal S6 kinase (RSK) pathway, the Mitogen- and stress-activated kinase 1 (MSK1)/CREB pathway, and the PKA/CREB pathway 34-36. The expression of EDNRB is regulated by MITF, suggesting EDNRB signaling and MITF expression form a self-reinforcing positive feedback loop that promotes melanocyte proliferation and melanogenesis 25. After wounding or UVR, Endothelin 1 (EDN1)/EDNRB signaling enhances the proliferation and differentiation of McSC and increases the regeneration of epidermal melanocytes 22, 35.

### PI3K/AKT

It is well-established that the phosphoinositide 3-kinase (PI3K) signaling pathway activates the serine/threonine-specific protein kinase (AKT) to increase GSK3β enzyme activity and prevent melanogenesis 37. MC1R can activate PI3K/AKT signaling to produce a negative feedback effect on melanogenesis and stimulate the extracellular release of melanin, preventing oxidative stress, DNA damage, and reduced survival 38. Although SCF/c-KIT is an upstream signal for PI3K/AKT activation in melanocyte and melanoma cells 39, 40, whether c-KIT has such a negative feedback loop remains to be demonstrated experimentally.

### TGF-β

Transforming growth factor-β (TGF-β) is the major regulator of McSCs maintenance, causing cell cycle arrest, downregulation of MITF and melanogenic genes, which ultimately keeps McSCs in a state of immaturity and quiescence 41. MITF and its downstream genes are the main targets of TGF-β in inhibiting melanogenesis 42-45. The TGF-β/Smads pathway negatively regulates the paired-box homeotic gene 3 (PAX3), which works synergistically with the SRY-Box Transcription Factor 10 (SOX10) to upregulate MITF, dependent on a cAMP-response element (CRE) 46, 47. Both TGF-β1 and TGF-β3 also downregulate MITF by activating ERK signaling to decrease melanogenesis 48, 49. Lastly, but importantly, TGF-β1 and TGF-β2 are recognized as key catagen-inducing growth factors of HF 50, 51. It has been demonstrated in melanoma cell lines that the direct transcriptional target of TGF-β1-the Kruppel-like transcription factor GLI2 not only suppresses MITF through PKA/cAMP signaling 52 but also directly downregulates tyrosinase-related protein 2 (TRP2) by competitive inhibition of CREB 53.

### MITF

MITF is a critical transcriptional regulator of melanogenesis and McSC maintenance and differentiation 26, 54. MITF positively regulates pigmentation-associated genes to promote differentiation-associated function and directly transactivates promoters of three primary melanogenesis enzymes, tyrosinase (TYR), tyrosine-related protein-1 (TYRP-1), and dopachrome tautomerase, also known as tyrosine-related protein-2, TYRP-2 (-2) 55. MITF also upregulates genes contributing to melanosome function, such as G Protein-Coupled Receptor 143 (GPR143), SILV, and melanoma-associated antigens recognized by T cells (MART-1) or melanosome transport such as Rab27 and Myosin5a (MYO5a) 55, 56.

Furthermore, MITF plays a role in melanocyte proliferation. The capacity of MITF to increase cyclin-dependent kinase 2 (CDK2) expression highlights its functions as a pro-proliferative factor 57. MITF also has been known to activate the transcription of T-box transcription factor 2 (TBX2) 58, which inhibits senescence by repression of p21 and p19 and participates in melanocyte growth and invasion 59, 60. Furthermore, MITF directly upregulates cell cyclin-related genes cyclin B1 (CCNB1) and cyclin D1 (CCND1) and mitotic genes such as polo-like kinase 1 (PLK1) 61.

Several downstream genes of MITF promote cell survival. MITF is the positive regulator of anti-apoptotic factors B-cell-lymphoma 2 (BCL2) and baculoviral IAP repeat containing 7 (BIRC7) 62, 63 and is involved in the regulation of the oncogenic hepatocyte growth factor receptor MET and the type III ribonuclease DICER, a necessary regulator of microRNA processing, thereby performing its anti-apoptotic effects 64, 65. It is widely thought that MITF mitigates DNA damage by increasing a group of repair genes, including DNA ligase I (LIG1), telomerase reverse transcriptase (TERT), essential meiotic endonuclease 1 homolog 1 (EME1), Breast Cancer 1 protein (BRCA1), and Fanconi anemia protein A (FANCA) 66. MITF transcriptionally regulates General transcription factor IIH subunit 1 (GTF2H1), which encodes the core component of Transcription Factor IIH (TFIIH), and CDK7, which encodes TFIIH kinase to promote the rapid recovery of nucleotide excision repair 67. In response to reactive oxygen species (ROS), MITF positively regulates apurinic-apyrimidinic endonuclease 1 (APE1), hypoxia-inducible factor 1 (HIF1α), and Peroxisome proliferator-activated receptor gamma coactivator 1-alpha (PGC1α) to improve survival capacity under oxidative stress 68-70.

It has recently been revealed that MITF enhances STIM1 expression transcriptionally 71, indicating the presence of a positive feedback loop between STIM1 and MITF to promote the MITF-inducing effect of MC1R or EDNRB signaling.

**Figure 1:**
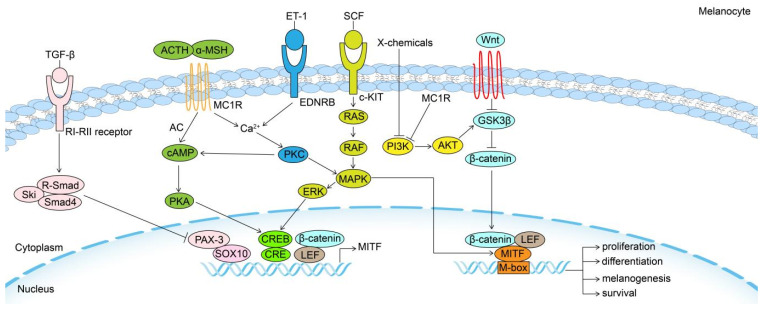
** Signaling pathways in the regulation of melanogenesis.** Melanogenesis and melanocytes proliferation, differentiation and survival are regulated by the MITF transcription factor, which is regulated by a number of important signaling pathways, including Wnt/β-catenin, KIT/SCF, ET-1/EDNRB, α-MSH/MC1R and TGF-β pathways. MITF: Melanocyte Inducing Transcription Factor; GSK3β: Glycogen synthase kinase 3β; AKT: serine/threonine-specific protein kinase; MC1R: Melanocortin 1 Receptor; TGF-β: Transforming growth factor-β; PI3K: phosphoinositide 3-kinase; SCF: stem cell factor; c-KIT: tyrosine kinase receptor; MAPK: mitogen-activated protein kinase; ERK: Extracellular signal-regulated kinase; ET-1: Endothelin 1; EDNRB: Endothelin receptor B; PKC: Protein kinase C; α-MSH: α-melanocyte-stimulating hormone; cAMP: cyclic adenosine monophosphate; PKA: cAMP-dependent protein kinase A; CREB: cAMP response element-binding protein; ACTH: adrenocorticotropic hormone; AC: adenylate cyclase; PAX3: paired-box homeotic gene 3; SOX10: SRY-Box Transcription Factor 10; CRE: cAMP-response element.

MITF can be regarded as a final common pathway in modulating the biological behavior of McSC and melanocyte to some extent, as several external and internal factors converge on it through different intracellular signaling pathways, and their effects depend on downstream genes (Fig. 1).

## Novel mechanisms of hair pigmentation regulation

### Sympathetic nerves and sensory nerves

It is widely acknowledged that HFs are innervated by sympathetic and sensory nerves 72. Under stressful conditions, these sympathetic nerves are hyperactivated and release noradrenaline in bursts, resulting in rapid McSC proliferation possibly mediated by β2 adrenergic receptors/AC/cAMP/PKA pathway. It has been shown that these sensory nerves can provide Sonic Hedgehog (SHH) signaling 73, which is required for the normal proliferation of melanocytes 74. Then these McSC differentiate ectopically and migrate out of the hair bulge, eventually causing the permanent depletion of McSC and irreversible grey hair 75.

### Neurotransmitters

Multiple neuropeptides released from intra-epidermal sensory nerves, such as calcitonin gene-related peptide (CGRP), substance P (SP), and vasoactive intestinal peptide (VIP), have been reported to regulate melanogenesis by acting on melanocytes directly or indirectly through immune and inflammatory responses 76, 77. In this context, we will primarily focus on their direct effects on HFs and melanocytes.

CGRP exerts catagen-inducing effects on human HFs and can maintain and restore hair follicle immune privilege (IP) via repression of MHC class I antigen 78, 79. Melanogenesis and melanocyte dendricity is enhanced by certain CGRP-induced keratinocyte-derived melanotrophic factors *ex vivo* 80. In mice, CGRP from sensory neurons increases insulin-like growth factor-I (IGF-I) production in the HF dermal papilla cells, thereby promoting hair growth and melanogenesis 81. However, not all studies support the conclusion that CGRP promotes melanin production. In this regard, it has been shown that in B16F10 cells, CGRP cooperates with SP to inhibit melanogenesis and induce cell apoptosis 82.

SP is released from sensory nerve endings induced by psychoemotional stress and regulates immune cells or HF mainly through neurokinin-1 receptors (NK1R) 83, 84. In organ-cultured HFs, SP upregulates nerve growth factor (NGF) and its apoptosis- and catagen-inducing receptor (p75NTR), while it downregulates the growth-inducing NGF receptor neurotrophic tyrosine kinase receptor type 1 (TrkA). Furthermore, MHC class I and β2-microglobulin are upregulated, suggesting that SP impairs immune privilege (IP) 84. Animal models have also shown that stress-induced SP not only inhibits HF keratinocyte proliferation and induces apoptosis but also promotes mast cell degranulation, causing the production of ROS and neurogenic inflammation, and then inhibits hair growth and induces the catagen phase in the hair cycle 83, 85, 86.

Studies have shown that in B10F16 melanoma cells, SP not only exerts a synergistic effect with CGRP, but also induces apoptosis in B10F16 cells and downregulates 5-hydroxytryptamine (5-HT)1A receptor and 5-HT2A receptor, both of which promote melanogenesis. This effect is mediated by binding to the NK1R receptor and activating S6 kinase 1 (S6K1) while inhibiting the MAPK signaling pathway 87-89. Moreover, excess SP induced by mental stress decreases melanogenesis through keratinocytes. During this process, the hypothalamic-pituitary-adrenocortical (HPA) axis is key in mediating the effects of SP signaling 90. However, contrasting studies revealed that SP promotes EDN1 secretion via endothelin-converting enzyme 1 and upregulates Wnt/ β-catenin signaling by downregulating the Wnt inhibitor Dickkopf-1 (DKK1) to increase melanogenesis in normal human melanocyte *ex vivo* 91, 92. Interestingly, the results from animal models present a paradoxical outcome. These contradictory results may be due to heterogeneity between cells and different concentrations of SP.

It is well-established that VIP, an immunoinhibitory neuropeptide secreted from perifollicular sensory nerve endings, prevents the HFs from IP collapse 93 and promotes melanogenesis in the B16F10 cell line and normal human melanocytes basically by activating the PKA/CREB/MITF signaling pathway 94.

### Adipose tissue

It has been shown that dermal white adipose tissue (dWAT) is located underneath and partially integrated into the reticular dermis, surrounding HFs 95. Interestingly, there is a strong interaction between HFs and dWAT 96. HFs drive a cycle of dWAT remodeling, where HFs secrete adipogenic activators at the beginning of new hair growth, and at the end of hair growth, activator secretion decreases or adipogenesis inhibitor secretion increases, leading to lipolysis 95, 97. dWAT is rich in growth factors that signal reciprocally to HF and regulates the activation state of their stem cells and the rate of hair regeneration 95.

Importantly, mature dWAT can inhibit hair growth. During early telogen, HFs enter a refractory phase to growth signaling, partly mediated by bone morphogenetic protein 2 (BMP2) expressed by adjacent dermal adipocytes 97. A large area of dWAT expresses BMP2, which highly maintains the quiescence of HFSC to prevent excessive hair production 95.

In contrast, dWAT in anagen positively affects hair growth. Adipose progenitor cell is essential for HF to enter a new anagen and is widely thought to stimulate telogen HFs through high levels of platelet‐derived growth factor alpha (PDGFA) 95. Hepatocyte growth factor (HGF), secreted by anagen perifollicular dWAT, stimulates Wnt/β-catenin signaling in the hair matrix by inhibiting Wnt antagonist frizzled-related protein 1 (SFRP1) as well as upregulating WNT10B, thereby promoting melanocyte maturation and pigmentation 98.

Current evidence suggests that dWAT plays significant roles in the aging process of HF 99. Aging dWAT represses Wnt signaling by upregulating Wnt inhibitors dickkopf-related protein (DDK) and SFRP4. Additionally, there is an increase in the expression of fibroblast growth factor (FGF)5 and BMP2, which inhibit hair growth, while the secretion of hair growth-promoting factors such as FGF10 and FGF7 is reduced. Compared with young dWAT, senescent dWAT in telogen expresses abundant abnormal inflammatory factors. In aging dWAT during anagen, the inflammatory process is largely suppressed, but collagen production, angiogenesis, and melanogenesis are impaired.

Given that melanogenesis and hair growth share common signaling pathways, it is highly conceivable that dWAT regulates hair graying, although further research is warranted to validate this hypothesis and explore the underlying mechanisms.

Adiponectin, an adipocyte hormone, is specifically and abundantly expressed in WAT. Adiponectin or activation of adiponectin receptor 1 (AdipoR1) can upregulate multiple hair growth factors through AMP-activated protein kinase (AMPK) in human follicular DPC, including IGF-1, vascular endothelial growth factor (VEGF), IGF, HGF, PDGFA, and FGF 7, and downregulate TGFβ1, thereby inducing the anagen and promoting hair growth *ex vivo* and *in vivo* 100-103. In contrast, *ex vivo* studies revealed that adiponectin oligomer downregulates pigmentation genes in HF and important factors such as Wnt10B and the HGF receptor c-Met within the hair matrix and DP 103. Meanwhile, neutralizing adiponectin isoforms within HF and dWAT promotes melanocyte proliferation, melanogenesis, and tyrosinase activity but produces fewer melanocytes and dendrites. Nevertheless, hair matrix keratinocyte proliferation and hair pigmentation were not altered by adiponectin oligomer within 48h *ex vivo*.

Adiponectin exists in two forms in circulation, a full-length protein and a fragment containing the globular domain of adiponectin (gAd). The full-length adiponectin induces depigmentation via AMPK/ CREB-regulated transcription coactivators (CRTCs)/CREB signaling pathway 104, but the gAd promotes melanogenesis by activation of the AMPK-p38 MAPK-CREB pathway 105. Hence, the proportion of adiponectin oligomer to globular adiponectin or HGF to adiponectin could determine the quantity of synthesized melanin.

Adipose-derived stem cells (ADSCs) are an important type of stem cell that can be isolated from adipose tissue. They are characterized by their ability to differentiate into multiple cell types, ease of availability, high proliferative capacity, and self-renewal potential 106. ADSCs exert their multiple regulatory roles mainly through autocrine and paracrine pathways 107.

Besides producing various cytokines that promote hair growth, such as VEGF, PDGF, and HGF 108, ADSCs also secrete exosomes to regulate HF. An increasing body of evidence suggests that adipose-derived stem cell exosome (ADSC-Exo) enhance DPC proliferation and survival and promote hair regeneration, mediated in part by inhibiting TGF-β/SMAD3 signaling by miR-122-5p carried in ADSC-Exo 109-111. Moreover, ADSC-induced amphiregulin promotes hair regeneration of skin-derived precursors (SKPs), a multipotent precursor cell population from the dermis capable of differentiating into several lineages through activation of PI3K and MAPK pathways 112. Stromal vascular fraction (SVF), the regenerative cell cocktail obtained mainly from ADSCs, has also been shown to treat alopecia areata effectively and safely 113.

In addition to promoting hair growth, ADSCs are also a regulator of melanogenesis. Interestingly, ADSCs can inhibit the proliferation and melanogenesis of epidermal melanocytes through an interleukin-6 (IL-6)-mediated mechanism 114 and through upregulation of TGF-β1 43. UVB-induced skin pigmentation is reduced in the area of the skin where ADSCs have been injected, possibly related to the fact that α-MSH/MCIR/cAMP signaling is suppressed by basic fibroblast growth factor (bFGF) secreted from ADSCs 115-117. It also has been reported that SVF can inhibit UVB-induced pigmentation in guinea pig skin 118.

Intriguingly, ADSCs have shown the potential to promote pigmentation in the context of vitiligo treatment. ADSCs not only promote the proliferation and migration of co-cultured melanocytes and reduce their differentiation 119 but also improve the effectiveness of melanocyte transplantation for vitiligo, likely because ADSCs upregulate bFGF and SCF and then increases the expression of integrins in melanocyte 120, 121. *Ex vivo*, adipose tissue extracellular fraction (AT-Ex) induces melanocyte intracellular antioxidant enzymes via acting on nuclear factor (erythroid-derived 2) -like (Nrf-2) to counteract oxidative stress, promotes cell proliferation, and inhibits GSK3β to activate Wnt/β-catenin signaling 122. Similarly, mice models revealed that NB UVB/ADSCs transplantation combination therapy could improve oxidative stress and calcium homeostasis by stimulating Nfr2/ heme oxygenase (HO -1) signaling, causing vitiligo repigmentation 123.

While there are some inconsistencies in the studies mentioned above, it is plausible to speculate that ADSCs may promote the proliferation and survival of melanocytes by reducing inflammation and maintaining melanocyte quiescence.

## Cases of gray hair repigmentation and possible mechanisms

### Therapeutic monoclonal antibodies

Monoclonal antibodies (mAbs), which are immunoglobulins, can target a specific epitope on an antigen and have emerged as an important class of therapeutic drugs. To date, numerous mAbs have received marketing approval 124.

A cell surface receptor called programmed death-1 (PD-1) acts as a T cell checkpoint and is critical in controlling T cell exhaustion. When PD-1 binds to its ligand programmed death ligand 1 (PD-L1), this triggers a downstream signaling pathway that suppresses T cell activation.

Tumor immune evasion is mediated by abnormally elevated PD-L1 expression on tumor cells and antigen-presenting cells in the tumor microenvironment 125. Anti-PD-1/PD-L1 antibodies, one of the immune checkpoint inhibitors (ICIs), restore the immune response to cancer cells by rescuing T cells from an exhausted state, which have been approved for treating multiple malignancies 126.

A series of 14 patients undergoing anti-PD1/anti-PD-L1 therapy for lung cancer demonstrated hair repigmentation, suggesting it is a promising indicator of positive treatment response 127. Besides, hair repigmentation of the entire body was reported in a male patient with concomitant advanced colorectal cancer and Hodgkin lymphoma who underwent nivolumab treatment, an anti-PD-1 antibody 128. It is widely thought that PD-1/PD-L1 immunotherapy and cytotoxic tumor destruction cause an inflammatory state, which results in the collapse of the immune privilege of HF and hair repigmentation 129. It has also been reported that melanogenesis-related genes and melanin production in B16F10 cells was downregulated by PD-L1 from polyinosinic-polycytidylic-treated HaCaT cell 130, which suggests the direct relationship between PD-1/PD-L1 and melanogenesis. However, vitiligo is a common immune-related adverse event in ICIs treatment for melanoma patients 131, possibly due to the induction of an anti-melanocyte response, which has not been observed in lung cancer patients 132. Moreover, positive staining of anti-PD-L1 antibodies was found in a canities subita patient who experienced extreme trauma 133. Considering these contradictory evidences, more research must be done to clarify the confusing relationship between melanogenesis and PD-1/PD-L1.

Dupilumab, a monoclonal antibody for interleukin 4 (IL-4) receptor alpha subunit, blocks the IL-4/IL-13/IL-4R axis and reduces T helper 2 (Th2) cell response effectively 134. Hair repigmentation has been reported in an atopic dermatitis patient treated with dupilumab 135. It has been revealed that IL-4 suppresses the expression of MITF, TYRP-1, and DCT through the Janus Kinase 2 (JAK2)/ Signal Transducer And Activator Of Transcription 6 (STAT6) signaling pathway and then inhibits melanogenesis in human normal melanocytes (HNMs) 136.

As a tumor necrosis factor (TNF) inhibitor, adalimumab is indicated to treat inflammatory disorders, including psoriasis, rheumatoid arthritis, and inflammatory bowel disease. There has been a reported case of hair repigmentation in a rheumatoid arthritis patient treated with adalimumab 137.

TNF inhibits melanogenesis and the viability of melanocytes through multiple pathways and is intricately connected with the pathogenesis of vitiligo 138. It has been revealed that both TNF and IL-17 treatment of melanocytes downregulated c-KIT, MC1R, MITF, DCT, and other melanogenesis-related genes, and the levels of tyrosinase and melanin significantly decreased 139. The combined action of TNF and IL-17 has been shown to inhibit melanogenesis through the PKA and MAPK signaling pathways 140. TNF and IL-17 have been found to induce the expression of β-defensin 3 in cultured keratinocytes. Acting as an antagonist for MC1R, β-defensin 3 can inhibit the activation of adenylate cyclase and tyrosinase induced by α-MSH 139. Other *in vitro* studies reveal that TNF-α could reduce melanocyte-stimulating hormones receptor (MSH-R) binding activity, MC1R expression, and the expression of a melanosomal protein gp87, promoters of melanogenesis 141, 142. IL-6, an inhibitor of melanogenesis, was significantly elevated in human normal human melanocytes treated with TNF-α, IL-17, and interferon-gamma (IFN-γ) 136, 138. *In vitro*, IL-6 decreases melanogenesis by reducing the transcription of MITF in melanocytes 143, 144 and blocking the paracrine function of keratinocytes and fibroblasts through the IL-6 / STAT3 / FGF2 pathway 145.

*In vitro*, the expression of intracellular adhesion molecule-1 (ICAM-1) in melanocytes can be upregulated by TNF-α, which may promote T cell/melanocyte adherence and immunologic cytotoxic damage, resulting in vitiligo 138, 146. Ample evidence suggests that TNF-α treatment increases ROS in melanocytes and leads to melanocyte toxicity 138, 146.

**Figure 2:**
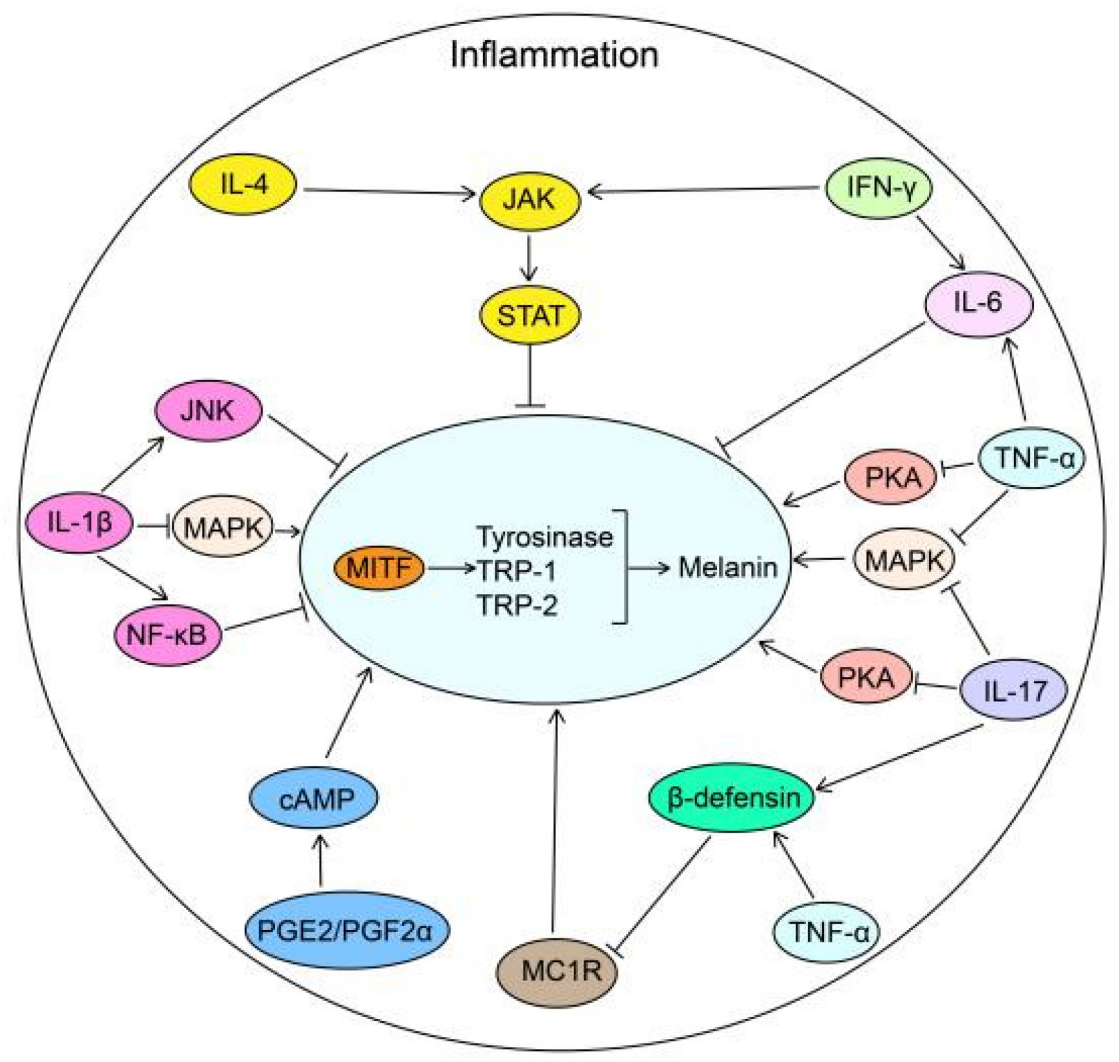
** The biological roles of inflammatory factors in melanogenesis.** Inflammatory factors including PGE2 and PGF2α promote melanogenesis by stimulating cAMP pathway. While IL-4 and IFN-γ inhibit melanogenesis through JAK-STAT pathway. TNF-α inhibits melanogenesis by suppressing the PKA, MAPK and MC1R pathways in combination with IL-17. IL-1β inhibits melanogenesis through NF-κB, JNK and MAPK pathways. IL-6, elevated via TNF-α and IFN-γ, decreases melanogenesis by reducing the transcription of MITF in melanocytes. IL-4: interleukin 4; JAK: Janus Kinase; STAT: Signal Transducer And Activator Of Transcription; IFN-γ: Interferon gamma; IL-6: interleukin 6; PKA: cAMP-dependent protein kinase A; MAPK: mitogen-activated protein kinase; IL-17: interleukin 17; TNF-α: tumor necrosis factor alpha; MC1R: Melanocortin 1 Receptor; PGF2α: Prostaglandin F2α; PGE2: prostaglandin E2; cAMP: cyclic adenosine monophosphate; NF-κB: nuclear factor-κB; JNK: c-Jun N-terminal kinase; IL-1β: interleukin-1β; MITF: Melanocyte Inducing Transcription Factor; TRP-1: Tyrosinase related protein-1; TRP-2: Tyrosinase related protein-2.

However, due to the high complexity of the body, TNF may have dual effects on melanogenesis. There is an increasing consensus that TNF-α can stimulate endothelins (EDNs) and SCF secretion from melanocytes and keratinocytes to cause skin hyperpigmentation 147-149. Additionally, TNF-α-induced production of reactive oxygen species (ROS) contributes to melanogenesis 150. It is widely thought that Adalimumab may mitigate the high level of TNF-α in rheumatoid arthritis patients, resulting in hair repigmentation, but the underlying mechanism warrants further research (Fig. 2).

Hair pigmentation was observed in a patient with plaque psoriasis undergoing treatment with secukinumab 151. Secukinumab is a fully human monoclonal antibody that targets interleukin-17A and is utilized in the treatment of various autoimmune diseases.

In addition to its synergistic effects with TNF, IL-17 suppresses melanogenesis through ROS-dependent autophagic melanocyte apoptosis and stimulates keratinocytes to secrete IL-1β through the nuclear factor-κB (NF-κB)/ROS/ NLR Family Pyrin Domain Containing 3 (NLRP3)/caspase 1 pathway 152. Similarly, the production of TNF-α and IL-6 in keratinocytes and fibroblasts is increased dramatically by IL-17A *ex vivo* 153. Further investigation is needed to determine the actual impact of IL-1β on melanogenesis when it directly interacts with melanocytes. In melanoma cell lines, IL-1β has been shown to downregulate MITF expression through the activation of NF-κB, c-Jun N-terminal kinases (JNKs), and microRNA-155 154, 155. Additionally, IL-1β has the ability to induce apoptosis in melanocytes 156. However, contrasting findings have also been observed. It has been discovered that IL-1β can upregulate MC1R expression in normal human melanocytes 142. Furthermore, recent research has indicated that IL-1β partially mediates UVB-induced melanogenesis by upregulating the expression of TYR and TRP1 in melanoma B16 cells 157. Nevertheless, it is unquestionable that both IL-17 and IL-1β are positively associated with the progression and extent of vitiligo, likely due to autoimmune melanocyte death 158-161.

Interestingly, an increase in melanocyte abundance and a concomitant decline in pigmentation signaling was observed in psoriasis lesions known to overexpress IL-17 and TNF 139. It has been reported that speckled lentigines appeared in resolved psoriatic plaques after treatment with biological agents, such as scukinumab^162^, ^163^. The occurrence of lentigines may be attributed to the removal of inhibition on melanogenesis when TNF-α and IL-17 are blocked. When these inhibitory factors are suppressed, melanocytes in resolved lesions may produce excessive melanin, leading to the formation of lentigines. The mechanism of hair repigmentation following treatment may share similarities with the development of skin lentigines.

Ustekinumab, an anti-interleukin IL-12/23 p40 monoclonal antibody, induced hair repigmentation in a psoriasis vulgaris patient 164. It is widely believed that activation of the TH17 pathway by IL-23 is the predominant mechanism involved in the pathogenesis of psoriasis. The survival and proliferation of TH17 and TH22 cells depend on IL-23. IL-17, IL-22, and TNF-α are produced by TH17 cells, and IL-22 is produced by TH22 cells. Naive T cells are converted into TH1 cells by IL-12, which secretes IFN-γ and TNF-α 165. Ustekinumab may lead to hair pigmentation by decreasing these inhibitors of melanogenesis (IL-17 and TNF-α).

Brentuximab vedotin is an antibody-drug combination commonly used to treat CD30+ lymphomas. This conjugation binds to the CD30 antigen on the surface of cells expressing CD30. Upon absorption by the cell, the compound undergoes proteolytic cleavage, releasing monomethyl auristatin E. Interestingly, a patient who underwent allogeneic hematopoietic stem cell transplantation and received brentuximab vedotin as a trial for refractory chronic graft-versus-host disease (cGVHD) experienced hair repigmentation 166. CD30 signaling enhances the activation of TH1 and TH17 cells, leading to increased production of INF-γ and IL-17A. Additionally, CD30 signaling promotes the secretion of TNF-α and IL-6 through the activation of NF-κB 167, 168. Importantly, these cytokines (INF-γ, IL-17A, TNF-α, and IL-6) are known to negatively regulate melanogenesis. The observed hair repigmentation induced by brentuximab may be attributed to the elimination of these proinflammatory cytokines, which allows for the restoration of normal melanogenesis processes.

### Tyrosine kinase inhibitors

In a retrospective cohort study involving 133 chronic myeloid leukemia (CML) patients treated with imatinib, 9 cases of hair repigmentation were reported 169. Hair repigmentation has also been observed in a nilotinib-treated CML patient 170. Several cases of hair pigmentation following sorafenib and erlotinib treatment for lung adenocarcinoma have been documented 171, 172. Indeed, all these drugs belong to the class of tyrosine kinase inhibitors.

As the first protein tyrosine kinase inhibitor, imatinib inhibits Bcr-Abl, platelet-derived growth factor receptor (PDGFR), and c-Kit. It has been approved for treating Philadelphia-chromosome-positive CML and gastrointestinal stromal tumors (GIST) 173. Nilotinib and dasatinib are second-generation inhibitors developed to address imatinib resistance in CML 174. In recent years, alternative mechanisms of nilotinib have been discovered, such inactivation of p38 MAPK in microglial/astroglial cells and a myoblast cell line 175, 176, inhibition of the discoidin domain receptor (DDR) in metastatic colorectal cancer cells 177, and prevention of NF-κB activation in microglial cells 178 indicating there are more target points of nilotinib. It has been reported that nilotinib and dasatinib promote melanogenesis *in vitro*. In HM3KO melanoma cells, nilotinib was found to upregulate MITF and its downstream genes by activation of the cAMP/PKA/CREB signaling pathway and decreasing the phosphorylation of AKT, which repressed the pigmentation process by inhibition of GSK3β 179. In B16F0 mouse melanoma cells, nilotinib was found to increase ROS levels and ROS-induced JNK activation, thereby inducing TYR, TRP1, and TRP2 180. Dasatinib has also been discovered to promote melanogenesis in human normal melanocytes through ERK/CREB/MITF signaling and possibly through phosphorylation of p38 MAPK and JNK 181.

Sorafenib is a multiple-target tyrosine kinase inhibitor, inhibiting Raf1, VEGF receptors, platelet-derived growth factor (PDGF) receptors, and several other targets 182. It has been shown that Sorafenib also upregulates MITF and melanogenesis in the HM3KO melanoma cell line by repression of AKT and ERK pathway and increase of β-catenin via reduction enzyme activity of GSK3β 183.

It has been established that erlotinib selectively inhibits epidermal growth factor receptor (EGFR) and can be used to treat several solid tumors 184. Although EGFR signaling negatively affects UVR-induced melanogenesis 185, 186, it is highly conceivable that post-inflammatory hyperpigmentation is caused by erlotinib-induced follicle inflammation 171.

It has been established that tyrosine kinase inhibitors (TKIs) have various cutaneous adverse effects, with skin and hair depigmentation being relatively common but hair repigmentation occurring less frequently 187-189. The exact mechanism by which TKIs promote hair repigmentation is not yet fully understood, although it is believed to be due in part to their ability to enhance melanogenesis.

### Immunomodulatory drugs

Two cases have been reported in which multiple myeloma (MM) patients experienced hair repigmentation after receiving lenalidomide or thalidomide treatment 190, 191. These drugs, which are immunomodulatory drugs (IMiDs), are approved for specific types of hematological cancers and autoimmune diseases. Protein cereblon (CBRN) is a member of the Lon protease family that plays a crucial role in mediating the anti-myeloma effects and teratogenicity of this class of IMiDs 192. IMiDs have been shown to decrease the production of TNF-α in human peripheral blood mononuclear cells (PBMCs) by promoting the degradation of TNF-α mRNA, potentially through CBRN 192. Additionally, IMiDs have been demonstrated to reduce the expression and secretion of IL-6, which is known to be upregulated by TNF-α 193. IMiDs also produce therapeutic effects on several diseases by inhibiting TGF-β 194-198. It has also been reported that IMiDs reduce IL-1 and IL-1β in plasma 199. What's more, IMiDs inhibit the activation of NF-κB by blocking the degradation of inhibitor of NF-κB (IκB) proteins 200.

TNF-α, IL-6, TGF-β, and IL-1β are all recognized as inhibitors of melanogenesis 201. The impact of TNF-α and IL-6 on melanogenesis was previously examined in the context of "Adalimumab," while the influence of IL-1β was discussed in relation to "Secukinumab."

Moreover, it has been established that IL-10, which activates the STAT-3 and PI3K/AKT/NF-κB signaling pathways to protect primary melanocytes 202, is induced by IMiDs 192, 203.

NF-κB signaling participates in both the stimulation and the suppression of melanogenesis. It was reported that TNF-α, tumor necrosis factor superfamily member 14 (TNFSF14), and IL-1β induce melanogenesis by activating NF-κB 204, 205. However, other studies suggest that activation of NF-κB mediates the facilitation of melanogenic activity from multiple sources, such as IL-18, Toll-like receptor 9 agonists, and UVR-induced-oxidative stress, indicating that the target genes involved in regulating melanogenesis may not be identical when NF-κB signaling is activated 206-208.

Interestingly, in multiple myeloma patients, IMiDs increase the production of IFN-γ (an inhibitor of melanogenesis) from T cells, and IMiDs downregulate VEGF, bFGF, and granulocyte macrophage-colony stimulating factor (GM-CSF), all of which have promotive effects on melanogenesis 193, 201, 209-213. The hair repigmentation effects of IMiDs are primarily attributed to their ability to inhibit the elevated levels of melanogenic inhibitors observed in multiple myeloma patients.

### Cyclosporine A (CsA)

Cyclosporine A, the first reported immunosuppressive drug to selectively inhibit T cells, mainly targets Th cells to achieve its therapeutic effect 214. CsA has been shown to induce hair repigmentation in psoriasis patients 215-217. HF is an immediate target of CsA, and hypertrichosis may be the most intriguing and most common adverse effect of CsA 218. *In vitro*, CsA downregulates SFRP1 in DP, an inhibitor of the Wnt ligand, which activates the Wnt/β-catenin pathway in HF and then induces hair growth 219. Therefore, cyclosporine may promote melanogenesis by activating the WNT pathway in melanocytes. However, it is possible that CsA's immunosuppressive properties and its ability to reduce cytokine levels could contribute to hair repigmentation by inhibiting melanogenesis.

### Other drugs

A study revealed that hair repigmentation occurred in two patients (one case is myxedema coma, and the other is iatrogenic hyperthyroidism) after receiving high-dose thyroxine treatment 220. In organ-cultured normal human scalp HFs, TH enhances the proliferation of hair matrix keratinocytes, inhibits their apoptosis, and induces and prolongs the anagen phase through the downregulation of TGF-β2, a key catagen-promoting growth factor 220, 221.

It has been reported that T4 downregulates the intrafollicular expression of clock genes (BMAL1 and PER1) after 24h, both of which inhibit anagen/prolong catagen and inhibit HF pigmentation 222. Both T3 and T4 significantly promote melanogenesis of organ-cultured HF, the mechanism of which possibly is independent of the hair cycle 221. Thyroid hormone signaling is related to many pigmented dermatoses and hair disorders, such as vitiligo, melanocytic nevi, and alopecia areata 223, 224, indicating that thyroxine plays an important role in hair pigmentation and maintaining the homeostasis of melanocytes.

Hair repigmentation was induced in a glaucoma patient treated with latanoprost, a prostaglandin F2α (PGF2α) analog 225. It is now understood that iris pigmentation, eyelashes hypertrichosis, and hyperpigmentation are common adverse events 226. It has been revealed that PGF2α and prostaglandin E2 (PGE2) promote melanocyte dendrites formation and the activation of tyrosinase through cAMP/PLC signaling 227-230. Moreover, *ex vivo*, PGE2 promotes the delivery of filopodia and quantities of shedding spheroid granules in melanocytes (MCs) but does not influence the morphology of keratinocytes 231. Current evidence suggests that local application of latanoprost activates HFs and encourages hair growth, and bimatoprost, another PGF2α analog, has been approved for treating eyelash hypotrichosis 232, 233. A murine model validated the stimulatory effect of PGF2α and latanoprost on follicular melanogenesis and hair regrowth 234.

A retrospective study reported that hair repigmentation occurred in 24 of the 62 Alzheimer's patients receiving prolonged cholinesterase inhibitor therapy 235. Solar light not only induces skin keratinocytes to secrete acetylcholine (ACh), which represses light-induced melanogenesis probably by inhibiting cAMP/CREB/MITF signaling in melanocytes 236, 237 but also promotes expression of AChE in keratinocytes through transcription factor activator protein 1 (AP1) *ex vivo* 238. What's more, during melanin production in melanocytes and B16F10 melanoma cells, AChE is downregulated by increased cAMP/CREB signaling 237. The above findings suggest that ACh and AChE inhibitors are local negative regulators of melanogenesis, and there is a negative feedback loop of ACh-melanogenesis-AChE among melanocytes and keratinocytes to maintain melanin homeostasis in the skin.

Nevertheless, it was recently reported that phagocytosis mediated by the α7 nicotinic acetylcholine receptor causes the skin keratinocyte to take up melanosomes in response to UV exposure *in vitro* 239. Moreover, M4 receptor-KO mice exhibit poor hair growth with no HF melanogenesis 240, indicating that cholinergic signaling is indispensable for pigmentation.

It has been established that these AChE inhibitors work by increasing ACh in the nervous system to treat patients with Alzheimer's disease. Several reports revealed that ACh enhances the release of α-MSH from pituitary melanotropes and nerve-induced pigmentation in lower vertebrates 241. In a similar vein, it is also possible that ACh causes hair repigmentation in a neuroendocrine-dependent manner. Meanwhile, considering cholinergic signaling has multiple effects on neural and intestinal stem cells 242, ACh likely plays a role in McSC homeostasis.

A case report has documented that hair repigmentation occurred in a breast cancer patient who received treatment with tamoxifen, the first selective estrogen receptor modulator, for 2.5 years 243. Although there are conflicting reports, estrogen is considered to have ER-mediated promotive effects on melanogenesis in melanocyte *ex vivo*, 244 possibly by activation of the cAMP/PKA/MITF pathway 245, 246. Estrogen also induces melanogenesis indirectly via keratinocytes 244. *In vivo*, high estrogen levels enhance melanogenesis and cause skin hyperpigmentation, such as melasma 247. However, *in vitro*, tamoxifen was found to stimulate synthesis and extrusion of melanin, with decreased cAMP but upregulated catalase expression in normal human melanocytes, suggesting tamoxifen has a ROS-mediated promelanogenic effect on melanocytes 248. In practical terms, one of the established anti-cancer mechanisms of tamoxifen involves increasing apoptosis through the generation of reactive oxygen species 249.

L-DOPA has been reported to induce hair pigmentation in 3 patients with Parkinson's disease 250. In addition to being the intermediate of melanogenesis, L-DOPA is also a bioregulatory molecule that positively regulates melanogenesis by upregulating TYR and MC1R and regulating several cellular processes, such as cellular metabolism 251. These can be mediated by interacting with certain receptors or by non-receptor mechanisms.

Nevertheless, a study revealed that dopamine directly induced catagen in human scalp HFs *ex vivo* 252. Thus, L-DOPA may not be a good choice for beauty lovers who desire pigmented hair.

Cerebrolysin is a low-molecular-weight neuropeptide obtained from the porcine brain and has neuroprotective and neurotrophic effects similar to neurotrophic growth factors. It has been reported that cerebrolysin causes hair repigmentation linked to Melan-A, also known as MART-1 reactivation, in 5 neurological patients 253. Besides, some neuropeptides, such as CGRP, SP, and VIP, enhance melanocyte proliferation and melanogenesis 80, 91, 94. In addition, the p75 neurotrophin receptor (p75NTR) regulates apoptosis in the external root sheath of the HF 254. Therefore, cerebrolysin may cause hair repigmentation as a neurotrophin factor. Meanwhile, given that melanocytes are derived from the neural crest, the neuroprotective effects of cerebrolysin, which include preventing nerve cell apoptosis and promoting differentiation and migration, may also be beneficial for melanocytes 253.

As second-generation retinoids, etretinate and acitretin exert their effects by binding to retinoic acid receptors and retinoid-X receptors. Interestingly, in a study involving four patients, both etretinate and acitretin were found to induce hair repigmentation and curling 255-258. It has been demonstrated that an increased level of retinoic acid (RA) upregulates the C-KIT receptor and then sensitizes McSCs to KIT-ligand, eventually leading to ectopic McSCs differentiation in the niche *in vivo* 19. Although the mechanism behind hair color change induced by etretinate and acitretin is not completely understood, it is possible that their impact on retinoic acid metabolism could be an alternate mechanism. However, current research only suggests that etretinate and acitretin increase the telogen phase of the hair cycle and inhibit melanocyte proliferation through retinoid-X receptor signaling 259, 260.

According to some reports, some plant extracts have ability to prevent hair graying. *Eriodictyon angustifolium* (Ea) is a plant that grows on the west coast of North America and has been used for many years as a traditional medicinal herb by the indigenous population. Abundant flavonoids contained in Ea extract, such as sterubin and hydroxygenkwanin, seem to be active ingredients to prevent and reduce hair graying 261, 262. Although the target is not molecularly clear, sterubin appears to function by activating Wnt signal and reducing ROS 263, while hydroxygenkwanin relies on KIT signaling 264. *Polygonum multiflorum*(PM) is a tranditonal Chinese medicine that has been experiencely used to treat early graying hair for a long time. PM extract has been proven to potentiate melanin synthesis by targeting on α-MSH and MC1R and reducing ROS production 265-267. Besides Ea and PM, *Pueraria lobata* extract and its active compound, puerarin, also have been reported to prevent hair graying via the cAMP/MITF signaling pathway 268, 269.

### Micro-injury

Hair repigmentation was observed in an 84-year-old woman after Mohs micrographic surgery and secondary intention wound healing 270. Physical therapy, such as phototherapy and microneedle, has been used to treat pigmentation disorders and hair loss for years 271-273. In response to injury or UVB, HF-McSCs migrate to the epidermis, depending on MC1R signaling. Then they differentiate to produce protective pigmentation against UV 30. Except for the absence of pigmentation, *de novo* regenerated hair follicles can't be distinguished from regularly growing hair follicles. However, when mice are injured during the anagen, *de novo* regeneration of pigmented hair is seen. This phenomenon may be caused by the fact that, stimulated by increased Wnt7a of the keratinocytes, McSCs that have been induced to migrate to the interfollicular epidermis by injury are integrated into *de novo* regenerated hair follicles 274. Moreover, activation of the β-Catenin signaling pathway, at least in part, in melanocyte stem cells located in the hair follicle bulge area is responsible for the narrowband UVB (NBUVB)-induced repigmentation of vitiligo 275. Recent studies have also reported that the Wnt/β-Catenin pathway is involved in hair regeneration and vitiligo repigmentation following micro-injury 276.

As a form of injury, epilation activates McSCs to regenerate follicular and epidermal melanocytes through induced endothelin 3/endothelin type-B receptor (EDN3/EDNRB) signaling, leading to skin and hair hyperpigmentation 277.

Overall, the mechanism of micro-injury-induced hair repigmentation is closely related to the Wnt/β-Catenin and EDN3/EDNRB pathways.

### Tumor

Roughly ten cases have been reported documenting focal hair repigmentation occurring on and around the area of scalp melanoma 278-287. A hypothesized model for this phenomenon involves two key cascades. The first cascade involves infiltrating melanoma cells, directly delivering melanin to follicular keratinocytes 283, 287. However, histopathological examination revealed that many hair follicles remain uninvaded by melanoma cells but still exhibit repigmented hair. This leads to the second cascade, where benign bulbar melanocytes are activated by paracrine factors (such as SCF) released by neighboring melanoma cells 283, 287. Immunostaining studies have confirmed the presence of TGF-β1-expressing follicular epithelium adjacent to highly TGF-β1-positive melanoma cells 287, supporting this second cascade.

It has been reported that paraneoplastic syndrome caused by lung cancer could induce the darkening of hair and skin in several patients. The potential mechanism may be that a high level of ACTH in the body promotes melanogenesis through the MC1R signaling pathway 1, 288 (Table 1).

## Conclusion

While widespread repigmentation of gray hair is uncommon, the underlying mechanism remains an important area of study. Understanding the regulatory mechanisms involved in hair follicle melanogenesis and melanocyte stem cells is essential for developing potential clinical therapies to reverse gray hair. However, achieving this goal necessitates extensive research efforts. Given that the skin is an accessible part of the body and the position of the HFs is relatively superficial, topical treatment will have great advantages in safety and effectiveness.

Just as topical minoxidil is commonly used to treat hair loss, topical agents that stimulate melanin synthesis to reverse gray hair will have promising prospects if they can be developed in the future. Physical therapies such as photodynamic therapy and microneedling are also viable options. However, all these treatments rely on the presence of a sufficient population of active McSCs. Therefore, maintaining a healthy population of McSCs is also an urgent problem that needs to be addressed.

## Figures and Tables

**Table 1:** Cases of gray hair repigmentation and possible mechanisms

Cases		possible mechanisms
Monoclonal antibody drug	Anti-PD-1/PD-L1 therapy	An inflammatory state caused by PD-1/PD-L1 immunotherapy and cytotoxic tumor destruction results in the collapse of the immune privilege of the hair follicle and promotes hair repigmentation.
	Dupilumab	It removes IL-4/JAK2/STAT6 signaling pathway's inhibition of melanogenesis.
	Adalimumab	It blocks TNF-α and IL-17 signaling and then gets rid of inhibition on melanogenesis.
	Scukinumab	It blocks TNF-α and IL-17 signaling and gets rid of inhibition on melanogenesis.
	Ustekinumab	It decreases TNF-α and IL-17 produced by TH17 and TH22 cells.
	Brentuximab	It blocks CD30 signaling in TH1 and TH17 and then decreased INF-γ, IL-17A, TNF-α and IL-6.
tyrosine kinase inhibitors	Nilotinib	It increases melanogenesis through activation of cAMP/PKA/CREB/MITF signaling pathway and decreases AKT signaling. It also elevates ROS and induces JNK activation to upregulate TYR, TRP-1, TRP-2.
	Dasatinib	It promotes melanogenesis through MAPK or JNK/ERK/CREB/MITF signaling.
	Imatinib	The reason may be similar to Nilotinib and Dasatinib's.
	Sorafenib	It represses AKT and ERK pathway and increases β-catenin.
	Erlotinib	Post-inflammatory hyperpigmentation after erlotinib-induced follicle inflammation.
Immunomodulatory drugs	lenalidomide or thalidomide	They decrease inhibitors of melanogenesis and increase promotors.
		
Immunosuppressant	Cyclosporine A	CsA downregulates SFRP1, an inhibitor of Wnt ligand, which in turn activates Wnt/β-catenin pathway and then induces hair repigmentation.
Other drugs	L-thyroxine	TH inhibits TGF-β2 and promotes melanogenesis. BMAL1 and PER1 are downregulated to prolong anagen.
	Latanoprost	PGE2 and PGF2α stimulate melanogenesis by cAMP/PLC signaling and promoting melanosome delivery in melanocyte. Promotes hair follicle growth.
	Acetylcholinesterase inhibitor	Not clear but cholinergic signaling is necessary for melanogenesis. Ach also seems to increase α-MSH secretion and regulate stem cells.
	Tamoxifen	It induces ROS in melanocyte to stimulate melanogenesis.
	L-DOPA	It upregulates TYP and MC1R to enhance melanogenesis.
	Cerebrolysin	As a kind of neuropeptide, it causes melanogenesis linked to Melan-A as a kind of neuropeptide
	Retinoids	The impact of etretinate and acitretin on RA metabolism, which upregulates C-KIT receptor and leads to ectopic McSCs differentiation, may be an alternate mechanism for hair color change
Mechanical stimulation	Micro-injury	It activates MC1R signaling, Wnt signaling and EDNRB signaling and then promotes melanogenesis.
Tumor	Scalp melanoma	Melanoma cells directly deliver melanin to keratinocytes or secrete some factors to activate surrounding normal melanocyte in a paracrine manner.
	Lung cancer	Paraneoplastic syndrome in lung cancer leads to high level of ACTH, which can stimulate MC1R signaling.

## Abbreviations

TKIs: tyrosine kinase inhibitors
HF: hair follicle
DOPA: dihydroxyphenylalanine
HFPU: hair follicle pigment unit
McSC: Melanocyte stem cell
ORS: outer root sheath
GSK3β: Glycogen synthase kinase 3β
MITF: Melanocyte Inducing Transcription Factor
EDNRB: Endothelin Receptor Type B
EpSCs: epithelial stem cells
TACs: transit-amplifying cells
MC1R: Melanocortin 1 Receptor
UVB: Ultraviolet B
POMC: pro-opiomelanocortin
α-MSH: α-melanocyte-stimulating hormone
ACTH: adrenocorticotropic hormone
cAMP: cyclic adenosine monophosphate
PKA: protein kinase A
CREB: cAMP responsive element-binding protein
SOCE: Store‐operated Ca2+ entry
ER: endoplasmic reticulum
STIM1: stromal interaction molecule 1
SOC: store-operated Ca2+
PLC: phospholipase C
IP3: inositol triphosphate 3
ADCY6: adenylyl cyclase 6
UVR: Ultraviolet radiation
SCF: stem cell factor
MAPK: mitogen-activated protein kinase
ERK: Extracellular signal-regulated kinase
RSK: ribosomal S6 kinase
MSK1: Mitogen- and stress-activated kinase 1
EDN1: Endothelin 1
PI3K: phosphoinositide 3-kinase
AKT: serine/threonine-specific protein kinase
TGF-β: Transforming growth factor-β
PAX3: paired-box homeotic gene 3
SOX10: SRY-Box Transcription Factor 10
CRE: cAMP-response element
TRP2: tyrosinase-related protein 2
TYR: tyrosinase
TYRP-1: tyrosine-related protein-1
DCT: dopachrome tautomerase, also known as tyrosine-related protein-2, TYRP-2
GPR143: Protein-Coupled Receptor 143
MART-1: Melanoma-associated antigen recognized by T cells
MYO5a: Myosin5a
CDK2: cyclin dependent kinase 2
TBX2: T-box transcription factor 2
CCNB1: cyclin B1
CCND1: cyclin D1
PLK1: polo-like kinase 1
BCL2: B-cell-lymphoma 2
BIRC7: baculoviral IAP repeat containing 7
LIG1: DNA ligase I
TERT: telomerase reverse transcriptase
EME1: essential meiotic endonuclease 1 homolog 1
BRCA1: Breast Cancer 1 protein
FANCA: Fanconi anemia protein A
GTF2H1: General transcription factor IIH subunit 1
TFIIH: transcription factor IIH
ROS: reactive oxygen species
APE1: apurinic-apyrimidinic endonuclease1
HIF1α: hypoxia-inducible factor 1
PGC1α: Peroxisome proliferator-activated receptor gamma coactivator 1-alpha
SHH: Sonic hedgehog
CGRP: calcitonin gene-related peptide
SP: substance P
VIP: vasoactive intestinal peptide
IP: immune privilege
IGF-I: insulin-like growth factor-I
NK1R: neurokinin-1 receptors
NGF: nerve growth factor
p75NTR: apoptosis- and catagen-inducing receptor
TrkA: tyrosine kinase receptor type 1
5-HT: 5-hydroxytryptamine
S6K1: S6 kinase 1
HPA: hypothalamic pituitary adrenocortical
DKK1: Dickkopf-1
NAS: N-Acetylserotonin
CRS: chronic restraint stress
CUMS: chronic unpredicted mild stress
GABA: Gamma-aminobutyric acid
DPC: dermal papilla cell
dWAT: Dermal white adipose tissue
BMP2: bone morphogenetic protein 2
PDGFA: platelet‐derived growth factor alpha
HGF: Hepatocyte growth factor
SFRP1: frizzled related protein 1
DDK: dickkopf related protein
FGF: fibroblast growth factor
AdipoR1: adiponectin receptor 1
AMPK: AMP-activated protein kinase
VEGF: vascular endothelial growth factor
gAd: globular domain of adiponectin
CRTCs: CREB-regulated transcription co-activators
ADSC-Exo: Adipose-derived stem cell exosome
SKPs: skin-derived precursors
SVF: Stromal vascular fraction
IL-6: interleukin-6
AT-Ex: adipose tissue extracellular fraction
mAbs: monoclonal antibodies
PD-1: programmed death-1
PD-L1: ligand programmed death ligand 1
ICIs: immune checkpoint inhibitors
IL-4: interleukin 4
Th2: T helper 2
EoE: eosinophilic esophagitis
JAK2: Janus Kinase 2
STAT6: Signal Transducer And Activator Of Transcription 6
HNMs: human normal melanocytes
TNF: tumor necrosis factor
MSH-R: melanocyte-stimulating hormones receptor
FGF2: Fibroblast growth factor 2
ICAM-1: intracellular adhesion molecule-1
EDNs: endothelins
NF-κB: nuclear factor-κB
NLRP3: NLR Family Pyrin Domain Containing 3
JNKs: c-Jun N-terminal kinases
cGVHD: chronic graft-versus-host disease
CML: chronic myeloid leukemia
PDGFR: platelet-derived growth factor receptor
GIST: gastrointestinal stromal tumor
DDR: discoidin domain receptor
VEGF: vascular endothelial growth factor
PDGF: platelet-derived growth factor
EGFR: epidermal growth factor receptor
IMiDs: immunomodulatory drugs
CBRN: lon protease Cereblon
PBMSs: peripheral blood mononuclear cells
CRBN: Cereblon
IκB: inhibitor of NF-κB
TNFSF14: tumor necrosis factor superfamily member 14
MM: multiple myeloma
bFGF: basic fibroblast growth factor
GM-CSF: granulocyte macrophage-colony stimulating factor
CsA: Cyclosporine A
INF-α: interferon-α
HFs: hair follicles
TH: thyroid hormone
PGF2α: Prostaglandin F2α
PGE2: prostaglandin E2
MCs: melanocytes
AChE: Acetylcholinesterase
Ach: Acetylcholine
AP1: activator protein 1
L-DOPA: Levodopa
p75NTR: p75 neurotrophin receptor
RA: retinoic acid
HF-McSCs: hair follicle melanocyte stem cells
EDN3/EDNRB: endothelin 3/endothelin type-B receptor
sHG: secondary hair germ
NBUVB: Narrowband UVB
c-KIT: tyrosine kinase receptor
